# An Overview of Antennal Esterases in Lepidoptera

**DOI:** 10.3389/fphys.2021.643281

**Published:** 2021-03-31

**Authors:** Ricardo Godoy, Juan Machuca, Herbert Venthur, Andrés Quiroz, Ana Mutis

**Affiliations:** ^1^Programa de Doctorado en Ciencias de Recursos Naturales, Universidad de La Frontera, Temuco, Chile; ^2^Centro de Investigación Biotecnológica Aplicada al Medio Ambiente (CIBAMA), Universidad de La Frontera, Temuco, Chile

**Keywords:** Lepidoptera, antennal esterases, transcriptomic, semiochemicals, inhibition, olfactory system

## Abstract

Lepidoptera are used as a model for the study of insect olfactory proteins. Among them, odorant degrading enzymes (ODEs), that degrade odorant molecules to maintain the sensitivity of antennae, have received less attention. In particular, antennal esterases (AEs; responsible for ester degradation) are crucial for intraspecific communication in Lepidoptera. Currently, transcriptomic and genomic studies have provided AEs in several species. However, efforts in gene annotation, classification, and functional assignment are still lacking. Therefore, we propose to combine evidence at evolutionary, structural, and functional level to update ODEs as well as key information into an easier classification, particularly of AEs. Finally, the kinetic parameters for putative inhibition of ODEs are discussed in terms of its role in future integrated pest management (IPM) strategies.

## Introduction

In insects, the detection and processing of chemical cues through olfaction is crucial for successful mating, avoidance of harmful compounds, and location of either oviposition sites or food sources (Choo et al., 2013; Li et al., 2018). For instance, pollinators need to find floral resources using a sophisticated system capable of detecting and distinguishing volatile organic compounds (VOCs) in a short timescale (below 500 ms; Rusch et al., 2016). Unlike other senses, such as touch, vision, or hearing, the use of VOCs by insects (e.g., aphids, beetles, flies, and moths) to communicate messages over relatively long distances is an advantage (El-Shafie and Romeno, 2017).

**Figure 1:**
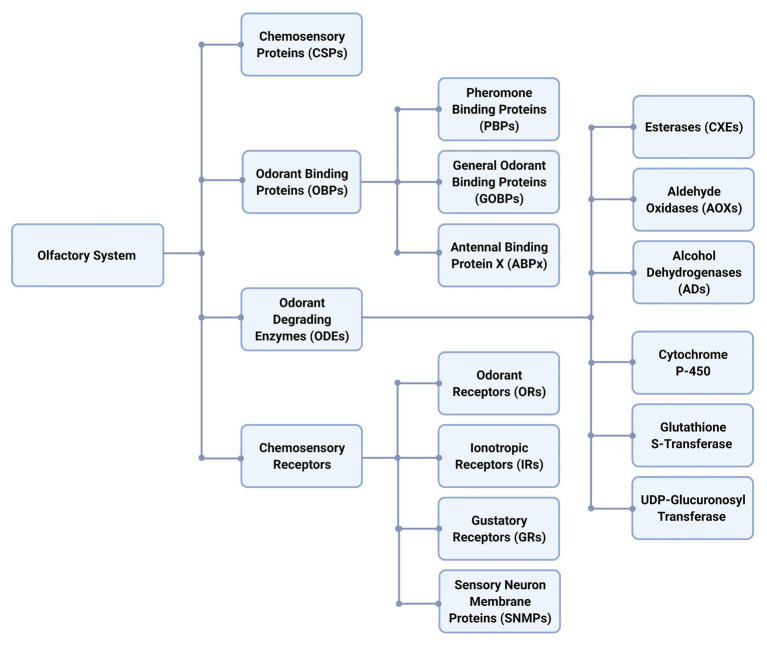
Schematic organization of proteins present in the olfactory system of Lepidoptera. It is possible to identify the four families, chemosensory proteins (CSPs), odorant-binding proteins (OBPs), odorant degrading enzymes (ODEs), and chemosensory receptors (Vogt, 2005; Pelosi et al., 2006, 2017; Rytz et al., 2013).

**Figure 2:**
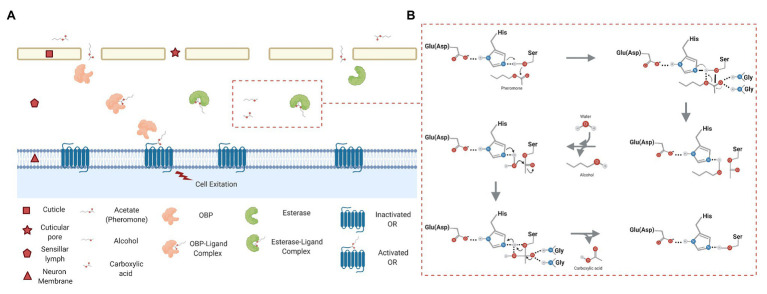
**(A)** Schematic representation of the olfactory mechanism in sensilla of Lepidoptera with emphasis on esterases. Compounds from the environment pass through cuticular pores toward the sensillar lymph. Here, OBPs bind and transport these molecules to odorant receptors (ORs) located in the dendritic membrane of olfactory neurons where they are activated. After cell excitation, the molecules are degraded by the action of ODEs (esterases). These enzymes can even act when the molecules enter to the sensillar lymph (Leal, 2013). **(B)** Reaction mechanism of the esterases in Lepidoptera. The ester hydrolysis occurs in a two-step reaction plus water addition. There is first a nucleophilic attack produced by the serine hydroxyl on the carbonyl carbon of the pheromone. The reaction is then stabilized by the histidine, and this amino acid is stabilized by the glutamic acid at the same time. A molecule of alcohol is then released, and the enzyme is acetylated. Second, the water molecule has affinity with the histidine residue and then acts as a nucleophile on the acetylated enzyme. Finally, a carboxylic acid is released, and the enzyme is free to start a new reaction. Importantly, there are two conserved glycines participating in the stabilization of the transition states in the oxyanion hole (Montella et al., 2012).

These volatile chemicals are called semiochemicals and mediate interactions between organisms of the same species (i.e., pheromone) and different species (i.e., allelochemicals). These chemicals are recognized through a systematic cascade of events that occur in chemosensory organs named sensilla, which can be found in maxillary palps, legs, and mainly covering the antennae (Zhou, 2010; Pelosi et al., 2017). Chemoreception is mainly related to four key protein families (Figure 1), such as odorant-binding proteins (OBPs), chemosensory proteins (CSPs), chemosensory receptors, and odorant degrading enzymes (ODEs; Pelosi et al., 2006, 2017; Mei et al., 2018; Song et al., 2018). Upon entry of odorants through cuticular pores, OBPs and CSPs transport these hydrophobic molecules across aqueous lymph (sensillar lymph) to odorant receptors (ORs). OBPs can be divided in three groups (Zhou, 2010): pheromone binding proteins (PBPs), general OBPs (GOBPs), and antennal binding protein x (ABPx; Krieger et al., 1996; Wang et al., 2020). The chemosensory receptors can be divided in ORs, ionotropic receptors (IRs), gustatory receptors (GRs), and sensory neuron membrane proteins (SNMPs; Klein, 1987; Pelosi and Maida, 1995; Isono and Morita, 2010; Zhou, 2010; Leal, 2013). Thus, a chemical signal is transduced into an electrical stimulus when ORs are activated (Sato and Touhara, 2008) leaving these semiochemicals in the sensillar lymph (Figure 2A).

One question that arises is the final destination of these chemicals after receptor activation: if these compounds are not degraded, then they could accumulate in the peripheral space interfering with the olfactory system in insects (Choo et al., 2013). Indeed, there are enzymes known as ODEs, which operate on the recovery of sensitivity in the olfactory system to detect new odorants (Sakurai et al., 2014; Zhang et al., 2017; Li et al., 2018).

Early studies reported the identification and isolation of an enzyme called antennae-specific esterase (ApolPDE) from the sensillar lumen of the Giant silk moth, *Antheraea polyphemus* (Vogt and Riddiford, 1981). Interestingly, through a kinetic study of ApolPDE, the authors showed that in the presence of this enzyme, the pheromone (*E*,*Z*)-6,11-hexadecadienyl acetate (E6,Z11-16:OAc) has an estimated half-life of 15 ms suggesting that ApolPDE is a pheromonal deactivator (Vogt et al., 1985). Ishida and Leal (2008) later supported this rapid degradation where ≈30 ms were necessary to reset the olfactory system of the Japanese beetle, *Popillia japonica*, through the study of the sex pheromone degradation using a recombinant enzyme. In this context, understanding the main actors in the inactivation of chemical signals is fundamental to the discovery of new molecules capable of disabling this mechanism (Leal, 2013). According to Vogt (2005), ODEs can be a target for behavioral inhibition because they degrade many different types of volatile compounds. Scientists have identified these enzymes as the beginning of more in-depth studies related to control and integrated pest management (IPM).

Despite the increasing amount of reported ODEs, no evolutionary analyses have been performed on these proteins among Lepidoptera. Furthermore, the structural features of the enzymes that could explain their selectivity have not yet been studied. Considering the diversity of acetate esters reported as sex pheromone compounds (463 acetate esters have been identified in the Pherobase database, https://www.pherobase.com/), the main focus of this review is the structure and evolutionary traits of antennal esterases (AEs) in Lepidoptera, which are responsible for the degradation of acetate ester-type pheromone components.

Thus, this text will offer a wider spectrum of new enzymes identified through bioinformatics techniques (i.e., transcriptomic) to attach a function through further functional studies. We propose specific guidelines that might help to clarify whether an ODE can be classified into a pheromone degrading enzyme (PDE) or not. The last step includes directed studies for this type of enzymes due to their participation in the degradation of pheromones, which in turn have a role in the behavior of organisms of the same species. In this way, new alternative and less costly pest management strategies could be implemented.

## Odorant Degrading Enzymes and Their Role in Sexual Communication

The olfactory system of insects has evolved to such an extent that it can process hundreds of compounds with different chemical structures from the environment to produce a change in behavior. Particularly, Lepidoptera emerged from a basal linage called non-Ditrysia to a new linage called Ditrysia since the Mesozoic era (over 100 million years); the species-specific pheromone components have similarly evolved. The sex pheromone that is generally emitted by females is crucial to attract a conspecific partner and achieve reproductive success. It is therefore not surprising that there are considerable structural differences in the blends of sex pheromones (Ando et al., 2004). We advise reviewing Löfstedt et al. (2016) for a complete understanding of the different types of pheromones in Lepidoptera.

Broadly, four groups of sex pheromones are described: Type 0 pheromones are structurally analogous to plant volatile compounds with short-chain ketones and alcohols. They are considered as primitive because they are also identified in non-Ditrysia species. The leaf miner moth, *Eriocrania semipurpurella*, is an example of non-Ditrysia moths and uses (2*S*, 6*Z*)-6-nonen-2-ol and (2*R*, 6*Z*)-6-nonen-2-ol as sex pheromone (Yuvaraj et al., 2017). Type I pheromones are biosynthesized *de novo* from acetate with C_10_–C_18_ alcohols, aldehydes, and esters. Type II pheromones are biosynthesized from decarboxylation and epoxidation from dietary linolenic or linoleic acids where C_17_–C_25_ polyunsaturated hydrocarbons are part of their structure (Millar, 2000). Type III pheromones contain one or more methyl branches in their structure with C_17_–C_23_ saturated and unsaturated hydrocarbons. Many sex pheromone compounds have been identified especially in Lepidoptera and other orders such as Diptera and Coleoptera (El-Sayed et al., 2006) since the discovery of (*E*,*Z*)-10,12-hexadecadien-1-ol (bombykol) – the sex pheromone of the silk moth *Bombyx mori* (Butenandt et al., 1959).

Most mating disruption techniques used today for controlling and monitoring moth pests are based on these chemicals (Arn et al., 1992; Witzgall et al., 2010). For instance, *Tuta absoluta* is a pest that attacks tomato crops, and components of its sexual pheromone [(*E*,*Z*,*Z*)-3,8,11-tetradecatrienyl acetate and (*E*,*Z*)-3,8-tetradecadienyl acetate] have been tested in greenhouses to control this insect (Cocco et al., 2012). Some studies have used inhibitors of ODEs such as trifluoromethyl ketones (TFMKs) to affect the pheromone detection and alter the behavior of moths. Malo et al. (2013) studied the male antennal response of the fall armyworm *Spodoptera frugiperda* against the inhibitor (*Z*)-9-tetradecenyl TFMK (Z9-14:TFMK) through electroantennography assay. This inhibitor is analogous to the main pheromone component (*Z*)-9-tetradecenyl acetate (Z9-14:Ac) of *S. frugiperda*, and it can significantly reduce the antennal response from 2.51 ± 0.37 mV to 1.10 ± 0.24 mV. On the other hand, Bau et al. (1999) disrupted the orientation flight of *Spodoptera littoralis* and *Sesamia nonagrioides* males in wind tunnel assays using TFMKs.

This background suggests that ODEs have an important role in the degradation of these pheromones. Consequently, one question that arises is if ODEs have evolved in order to degrade a wide range of these chemical cues or are limited to degrade a particular sex pheromone group for olfactory purposes. In this sense, transcriptomic analyses have provided the profile of ODEs that several moths could use for olfaction purposes. Acetates are the main sex pheromone components in Lepidoptera, and dozens of esterases in antennae have been reported as summarized in Table 1. Putative functions of ODEs have been suggested such as plant volatile and/or sex pheromone degradation though a few studies have actually addressed the functional role of some of these. In that sense, most enzymes characterized today are related to their sexual role in the degradation of pheromone components.

**Table 1:** Summary of esterases from Lepidoptera.

Lepidoptera species	Number of esterases	Technique of identification	Antennae-biased expression ^*^	Sex-biased expression	Esters in sex pheromone	Reference
*Antheraea polyphemus*	1 CXE	Native-PAGE	ApolPDE (Ant-spe)	M	E6,Z11-16:Ac	Vogt et al., 1985; Ishida and Leal, 2005
*Mamestra brassicae*	1 CXE	RACE-PCR	Mbra-EST (Ant-enr)	M = F	Z11-16:Ac	Maïbèche-Coisne et al., 2004
*Sesamia nonagrioides*	1 CXE	Native-PAGE and RACE-PCR	SnonCXE (Ant)	M = F	Z11-16:Ac	Merlin et al., 2007
*Sesamia inferens*	18 CXEs	Transcriptomic and RACE-PCR	4 SinfCXEs (13,14,18, and 20; Ant-enr)	M = F	Z11-16:Ac	Zhang et al., 2014
			SinfCXE10 (Ant-spe)	M = F		
*Spodoptera litura*	24 CXEs	Transcriptomic	4 SlitCXEs (Ant-enr)	M = F	Z9,E11-14:Ac; Z9,E12-14:Ac; E11-14:Ac; and Z9-14:Ac	Zhang et al., 2016
			SlitCXE10 (Ant-spe)	M = F		
*Spodoptera exigua*	6 CXEs	Transcriptomic and RACE-PCR	3 SexiCXEs (4, 17, and 20;Ant-enr)	M = F	Z9,E12-14:Ac; Z9-14:Ac	He et al., 2014
			3 SexiCXEs (5, 18, and 31; Ant)	M = F		
*Spodoptera littoralis*	20 CXEs	Native-PAGE,ESTs, and RACE-PCR	17 SlCXEs (Ant)	M = F	Z9,E11-14:Ac; Z9,E12-14:Ac	Merlin et al., 2007; Durand et al., 2010a,b, 2011
			SlCXE7 (Ant-spe)	M > F		
			SlCXE8 (Ant-spe)	M = F		
			SlCXE10 (Ant-enr)	M > F		
*Cnaphalocrosis medinalis*	18 CXEs	Transcriptomic	CmedCXE17 (Ant-enr)	M = F	Z11-16:Ac; Z13-18:Ac	Zhang et al., 2017
			CmedCXE20 (Ant-enr)	M = F		
			CmedCXE24 (Ant-enr)	M = F		
*Ostrinia furnacalis*	15 CXEs	Transcriptomic	–	–	E12-14:Ac; Z12-14:Ac	Yang et al., 2015
*Epiphyas postvittana*	5 CXEs	ESTs, 2D gel electrophoresis and mass spectrometry	–	–	E11-14:Ac; E9,E11-14:Ac; 12:Ac	Jordan et al., 2008
*Agrotis ipsilon*	17 CXEs	Transcriptomic	–	–	Z11-16:Ac; Z9-14:Ac; Z7-12:Ac; Z8-12:Ac; and Z5-10:Ac	Gu et al., 2013
*Plutella xylostella*	5 CXEs	Transcriptomic	5 PxylCXEs (Ant-enr)	M = F	Z11-16:Ac	He et al., 2017
*Ectropis obliqua*	35 CXEs	Transcriptomic	EoblCXE7 (Ant-enr)	M = F	^**^Z3,Z9-6,7-epo-18:Hy; Z3,Z6,Z9-18:Hy	Sun et al., 2017; Guo et al., 2019
			EoblCXE10 (Ant-enr)	M > F		
			EoblCXE13 (Ant-enr)	M = F		

PAGE, polyacrylamide gel electrophoresis; ESTs, expressed sequence tags; RACE, rapid amplification of cDNA ends; PCR, polymerase chain reaction; Ant, antennae; Ant-enr, antennae enriched expressed; Ant-spe, antennae specific expressed; Ant-sli, antennae slightly expressed; M, male; F, female; and (−), no information reported.

* The antennal-related location of esterases might not exclude other organs.

** No acetate ester-type as sex pheromone.

Transcriptomic advances have provided scientists a constantly growing database of sequences to identify ODEs including some with tissue-biased expression through reverse transcriptase polymerase chain reaction (RT-PCR) and quantitative (RT-qPCR) experiments. For example, 18 carboxylesterase (*CXE*) genes have been identified in the rice leaffolder, *Cnaphalocrocis medinalis*, through its antennal transcriptome (Zhang et al., 2017). Furthermore, the Egyptian armworm *S. littoralis* has an encoding-gene to *SlCXE7* that was 3-fold more expressed in males than females through RT-qPCR analysis (Durand et al., 2011). A greater expression of these enzymes in male antennae suggests that ODEs participate in the modulation of pheromone concentration since it is the female who produces and releases these semiochemicals to attract her conspecific mate in most species of moths.

## Evolutionary Traits of ODEs Across Lepidoptera

Insects are the most abundant and specious group of organisms. Nearly 150,000 species have been described in the Lepidoptera order alone. Considering their ecological impact, they have served as model systems to understand their mechanisms to locate mates and hosts plants as main examples. Furthermore, moths are important subjects of study within an evolutionary context due to their phenotypic plasticity, which comprises the ability of an organism (specifically a genotype) to respond to an environmental alteration with a change in its morphology, physiology, behavior, or life history (Moczek, 2010). These evolutionary processes are related to structural or regulatory mutations and change an amino acid in the coding region of a protein or affect the gene expression, respectively (Albre et al., 2012).

Genetic drift and natural selection can also contribute to the divergence of new enzymatic functions (Jones, 2017). In support of this, the cytochrome P450 enzymes (commonly involved in detoxification function) have had many mutations capable of catalyzing many chemical compounds (Bloom et al., 2007). In this sense, Lepidoptera are a clear representation of speciation where these changes are often related to their olfactory system for conspecific mate recognition. The extensive list of sex pheromone compounds identified to date (e.g., 463 acetate esters, 390 aldehydes, 331 primary alcohols, 299 secondary alcohols, and 28 tertiary alcohols reported in the Pherobase database) serves as a clue to the different types of enzymes involved in their biosynthesis. New desaturases have emerged by gene duplication and then diverged toward new functions (Roelofs et al., 2002). For instance, this has led to the evolution of castes and social organization in ants due to the expansion of the desaturase gene (Helmkampf et al., 2014). In this context, diverse enzymes are needed to degrade the large number of semiochemicals present in the environment that insects have to interact.

**Figure 3:**
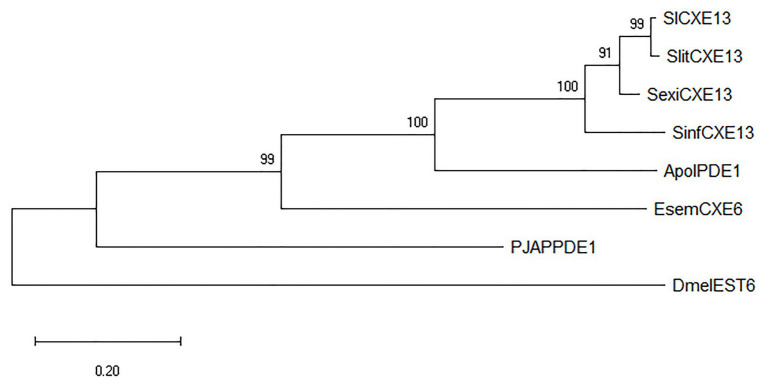
Phylogenetic tree of esterases. SlCXE13 (*S. littoralis*), SlitCXE13 (*S. litura*), SexiCXE13 (*S. exigua*), SinfCXE13 (*S. inferens*), ApolPDE1 (*A. polyphemus*), EsemCXE6 (*Eriocrania semipurpurella*), PJAPPDE1 (*Popillia japonica*), and DmelEST6 (*Drosophila melanogaster*). Phylogenetic analyses for esterases were performed by using MAFFT sever for multiple sequence alignments and FastTree software for phylogenetic relationships based on maximum-likelihood method (Price et al., 2010).

Odorant degrading enzymes have evolved from one gene family where catalytic and non-catalytic enzymes emerge (Oakeshott et al., 2005). Some studies have performed phylogenetic analysis for CXEs, and it is important to emphasize that the results showed a monophyletic clade where the most representative PDE (ApolPDE) was presented (He et al., 2014; Zhang et al., 2016). In Figure 3, we show a PDE clade (Supplementary Figure S1, a complete phylogenetic tree) constructed with some esterases in order to understand how these enzymes have evolved to degrade certain types of compounds. SlCXE13 from *S. littoralis* (Durand et al., 2010a); SlitCXE13 from *Spodoptera litura* (Zhang et al., 2016); SexiCXE13from *Spodoptera exigua* (He et al., 2014); SinfCXE13 from *Sesamia inferens* (Zhang et al., 2014); and ApolPDE1 from *A. polyphemus* (Ishida and Leal, 2005) belong to Ditrysia moths that use Type I sex pheromones. These enzymes are phylogenetically closer compared to EsemCXE6 from *E. semipurpurella*, a non-Ditrysia moth that use Type 0 sex pheromone. Moreover, PJAPPDE1 from *P. japonica*, and DmelEST6 from the fruit fly, *Drosophila melanogaster* were added due to their enzymes have been functionally well studied (Ishida and Leal, 2008; Younus et al., 2017). Lepidoptera, Coleoptera, and Diptera belong to the Holometabola group, but it is unknown the origin of physiological and morphological innovations in insects (Misof et al., 2014). Here, putative PDEs in Lepidoptera seem to have evolved from the ancient moth *E. semipurpurella*, as expected considering its non-Ditrysian origin. Overall, these results shed light on the evolution of these enzymes from *D. melanogaster*. It has been reported that DmelEST6 acts as an ODE with activity toward food esters, such as propyl, hexyl, heptyl, nonyl, decyl, neryl, and geranyl acetates (Younus et al., 2017). Therefore, it is proposed that Lepidoptera PDEs might have evolved from a common ancestor (i.e., DmelEST6), changing from catalytic activity toward food esters to specific acetate-based sex pheromone components. In this context, this issue could be considered when classifying an esterase as a PDE; however, a necessary functional assay to confirm such a role is needed.

## Antennal Esterases in Lepidoptera

Among insects, esterases have been classified in three major classes: (I) as enzymes with neuro/developmental functions; (II) intracellular enzymes with dietary detoxification functions, and (III) secreted enzymes that use hormones or pheromones as substrates (Claudianos et al., 2006). The latter is related to catalytically active enzymes that belong to *CXE* gene family and the α/β hydrolase superfamily (Punta et al., 2012). Their function is to hydrolyze esters in a two-step reaction plus water addition (Figure 2B). First, an alcohol-type metabolite is produced from the hydrolysis of ester bonds to subsequently generate an enzyme in an acylated, carbamylated, or phosphorylated form depending on its substrate (carboxylic, carbamic, or phosphoric ester, respectively). An acid-type metabolite is then formed and released due to water molecule addition so that the enzyme goes back to its active state (Montella et al., 2012). Thus, these CXEs play important roles, such as neurogenesis, development regulation, xenobiotic detoxification, and pheromones (Yu et al., 2009). The first CXE related to olfaction in moths was the PDE from *A. polyphemus* (Vogt et al., 1985); more recently, the CXEs identified in the tobacco cutworm *S. litura* (Zhang et al., 2016), the light brown apple moth *Epiphyas postvittana* (Jordan et al., 2008), and the black cutworm *Agrotis ipsilon* (Gu et al., 2013) among others have been studied by RNA-seq approaches (Table 1).

To date, *CXE* genes have been localized in different tissues of several moth species according to RT-PCR and RT-qPCR experiments. On the other hand, native polyacrylamide gel electrophoresis (Native-PAGE) has been used to study the enzymatic activity of some esterases (He et al., 2014). The expression in a specific tissue could shed lights on the function of a CXE. For example, Zhang et al. (2014) found five CXE genes in *S. inferens* where all of them (*SinfCXE10*, *SinfCXE13*, *SinfCXE14*, *SinfCXE18*, and *SinfCXE20*) were significantly expressed in different tissues, such as pheromone glands, thoraxes, abdomens, and antennae. However, *SinfCXE10* was expressed specifically in the antennae; therefore, the authors propose that this gene could be involved in pheromone degradation particularly in (*Z*)-11-hexadecenyl acetate. Although CXEs are present in olfactory organs (e.g., antennae), it seems that their pheromone-degrading function is more related to sex-biased expression. Durand et al. (2011) confirmed that a CXE gene from *S. littoralis* (*SlCXE7*) was restricted to antennae rather than other tissues through *in situ* hybridization (ISH) technique. This was more significantly expressed in males than in females according to RT-qPCR. Depending on its sensillar location such as pheromone-sensitive sensilla (i.e., trichodea, Str I), *SlCXE7* could play a role in pheromone signal termination as well as degrading odorant background noise.

On the contrary, He et al. (2014) reported one CXE gene from the beet armyworm, *S. exigua* (*SexiCXE4*), highly expressed in antennae and proboscises but had no sex-biased expression. *SexiCXE4* presented a higher preference (70-fold) to plant volatiles [(*Z*)-3-hexenyl acetate and hexyl acetate] than pheromone compounds (*Z*,*E*)-9,12-tetradecenyl acetate and (*Z*)-9-tetradecenyl acetate *via in vitro* functional assays. This suggests a role as a general ODE (GODE). Likewise, two CXEs genes (*EoblCXE7* and *EoblCXE13*) in the tea geometrid moth *Ectropis obliqua* were localized in pheromone-related sensilla (Str I) as well as sensilla basiconica (Sba) and gustatory sensilla styloconica (Sst) using fluorescence ISH (FISH) technique (Sun et al., 2017). *EoblCXE13* showed a differential expression pattern where it was restricted to the base of Str I in males. The lack of male-biased localization in this study suggests that the CXE genes might be involved in the hydrolysis of host plant volatiles rather than pheromone components (He et al., 2014). Despite the large amount of CXE expressed in the antennae of *E. obliqua*, no acetate ester-type sex pheromone degradation role could be attributed because this insect uses unsaturated hydrocarbons and enantiomers of epoxy hydrocarbons [(*Z*,*Z*,*Z*)-3,6,9-octadecatriene and 6,7-epoxy-(*Z*,*Z*)-3,9-octadecadiene] as sex pheromone (Type II sex pheromones; Sun et al., 2017). Likely, an epoxide hydrolase could participate in the degradation of the pheromone of *E. obliqua*, by catalyzing the hydrolysis of epoxide-like compounds to diols as many epoxy hydrolases do with epoxide-containing lipids (Morisseau, 2013), but further studies are needed.

## Structural Features of Esterases

The ability of proteins to bind to chemical compounds strongly depends on their amino acid constitution and conformation, such as domain arrangement, conformational dynamics, as well as the shape and amount of binding sites. Naturally, enzymes are not the exception with several enzymes as targets for the identification of substrates as well as competitive and non-competitive inhibitors or allosteric compounds in a drug-discovery approach.

A good example is the study of acetylcholinesterase (AChE) inhibitors such as organophosphorus compounds used as insecticides or toxic carbamates applied as pesticides (Čolović et al., 2013). For insects and particularly moths, evolution has provided highly specific adaptations for sexual communication that has resulted in evolved structural features. For instance, conserved structural regions have been found for ORs with seven transmembrane domains, increased sequence identity toward the C-terminal region, and, more interestingly, sequence motifs, such as LLLLECS, QQLIQ, and ILKTS in pheromone receptors (PRs; Zhang and Löfstedt, 2015; Köblös et al., 2018).

On the other hand, three and two conserved disulfide bridges play an important role giving structural stability in OBPs and CSPs, respectively (Pelosi et al., 2006). As a third player in perireceptor events, ODEs have not been structurally characterized so far from a wide viewpoint. In these cases, some enzymes appear to have preserved domains where acid (i.e., aspartic or glutamic acid) and base (i.e., histidine, arginine, or lysine) side chains of residues are part of the active sites (Jimenez-Morales et al., 2012).

After the identification of *A. polyphemus* CXE as a PDE (ApolPDE), structural investigations revealed that these enzymes share common features among moths. For instance, they have a conserved pentapeptide “G-X-S-X-G” (X represent any amino acid) motif that is characteristic of esterases (Cygler et al., 1993; Yin et al., 2011) and common catalytic triad “S-E(D)-H” that can catalyze the hydrolysis of esters – an important group of pheromone compounds with 10–18 carbon atoms and one or two unsaturated carbons (Löfstedt and Kozlov, 1997; Oakeshott et al., 1999). The absence of one of these residues causes these hydrolases to be transformed into catalytically inactive proteins (e.g., neurotactin or neuroligin); thus, they will be assigned in recognition or signal processing functions for neurodevelopment (Oakeshott et al., 2005). Moreover, CXEs bear an oxyanion hole formed with the amine group (–NH) from the “G-G-A” motif that stabilizes high-energy intermediates and the transition state through hydrogen bonding in the active site (Zhang et al., 2002, 2017). This feature is conserved in all esterase families in both vertebrates and invertebrates (Oakeshott et al., 1999).

On the other hand, some studies have found N-glycosylation sites that could help to improve resistance against proteolysis, reduce non-specific protein interactions, and increase the protein solubility and stability (Fonseca-Maldonado et al., 2013; Sun et al., 2015). The idea that CXEs are secreted into the extracellular medium is based on the presence of an N-terminal signal peptide (Oakeshott et al., 2005). This latter characteristic is relevant because they can be found in the sensillar lymph that can interact with the compounds that enter through the cuticular pores. Although CXEs do not share many similarities in the DNA sequences, they do have homology in their structure because the residues are conserved in the catalytic sites. Therefore, this family of proteins likely originated from a common ancestor (Nardini and Dijkstra, 1999).

**Figure 4:**
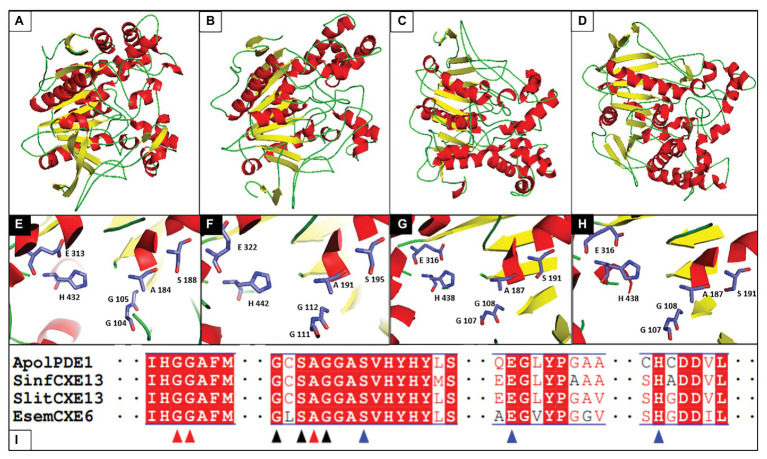
Modeled structures of esterases in Lepidoptera and partial alignment. **(A)** ApolPDE1 from *A. polyphemus*, **(B)** EsemCXE6 from *E. semipurpurella*, **(C)** SinfCXE13 from *S. inferens*, and **(D)** SlitCXE13 from *S. litura*. **(E–H)** Active sites of ApolPDE1, EsemCXE6, SinfCXE13, and SlitCXE13, respectively. **(I)** Partial alignment of amino acids sequences. Amino acids not shown are represented by two sequential dots. Oxyanion hole (G104-G105-A184) is indicated by red arrows. G181-X-S183-X-G185 motif is indicated by black arrows. The catalytic triad [S188-E(D)313-H432] is indicated by the blue arrows. The program Modeler 9.15 (Sali and Blundell, 1993; Webb and Sali, 2016) was used to build the three-dimensional structures and 4FNM (ApolPDE1), 5CH3 (SinfCXE13 and SlitCXE13), and 5THM (EsemCXE6) as templates were used to obtain these modeled structures. Moreover, molecular dynamic (MD) simulations were performed using the NAMD 2.9 (Phillips et al., 2005) so as to achieve a refinement of the modeled structure *via* the root mean square deviation (RMSD).

As mentioned in the previous sections, bioinformatic techniques have appeared as an alternative in the search for new enzymes. Thus, several antennal transcriptomes have been published and are very useful because they have free access. Figure 4 shows the modeled structures of the ApolPDE1 from *A. polyphemus*, EsemCXE6 from *E. semipurpurella* (public RNAseq raw data were downloaded from NCBI database, https://www.ncbi.nlm.nih.gov/, under the experiment SRX2627820), SinfCXE13 from *S. inferens*, and SlitCXE13 from *S. litura*. No crystallized CXEs structures have been published yet for Lepidoptera; however, three crystallized structures in Diptera with the access code 4FNM (Jackson et al., 2013), 5CH3 (Correy et al., 2016), and 5THM (Younus et al., 2017) in the protein data bank were used as templates in molecular modeling. These structures confirm the conservation of these enzymes in relation to their binding site regardless of their non-Ditrysia or Ditrysia origin.

SinfCXE13 and SlitCXE13 have been reported as enzymes expressed in the antenna, but they are not tissue-specific. Interestingly, these two are within the phylogenetic clade of enzymes secreted that use pheromones as substrates and are close to ApolPDE1 (He et al., 2014; Zhang et al., 2016). So far, putative esterase sequences from the transcriptome of *E. semipurpurella* have not been published; therefore, as a complement to this work, 17 CXEs transcripts were identified according to a phylogenetic analysis (data not included): Only EsemCXE6 was in the same clade as ApolPDE1, SinfCXE13, and SlitCXE13. Here, is possible to visualize the α-helices (red helical ribbons), β-sheets (yellow arrows), and loops (green smooth ropes) in all modeled structures (Figures 4A–D), which is consistent with those values reported for esterases (Montella et al., 2012). Interestingly, these characteristics are typical of α/β hydrolases and their folding gives a conformation of globular proteins. Furthermore, these sequences have a signal peptide indicating that these enzymes enter a secretory pathway, but they are not necessarily secreted to the extracellular environment (Nielsen, 2017). Despite the evolution of these moths, the antennal esterases conserved the residues in their active site that are responsible for the catalysis of chemical compounds (Figures 4E–H).

## Antennal Esterases Inhibition in Integrated Pest Management

Antennal esterases are ubiquitous in the olfaction process and can control the levels of stimuli in the sensilla through the rapid catabolism of semiochemicals-mainly acetate-type pheromones. Therefore, inhibition of these enzymes emerges as a complement to IPM because current strategies are costly, e.g., the use of synthetic pheromones for mating disruption (Guerrero and Rosell, 2005). Several analogous compounds to sex pheromones have been tested in Lepidoptera as reported by Reddy and Guerrero (2010), where TFMKs appear to be good candidates for enzyme inhibition. These molecules enter through cuticular pores (Figure 2A) toward the sensillar lymph where OBPs can bind these inhibitors. Indeed, Campanacci et al. (1999) showed that the (*Z*)-11-hexadecenyl TFMK was efficient in displacing the sex pheromone by binding to the recombinant PBP from *Mamestra brassicae* (MbraPBP1) through functional assays. Likewise, TFMKs can interact with ODEs inside the sensilla where it may form stable hydrates acting as transition-state analogues of pheromones. In brief, the inhibitory activity of TFMKs is due to a stable hemiacetal of tetrahedral geometry that is formed between the conserve serine residues of the antennal esterases with the highly electrophilic carbonyl (Durán et al., 1993; Wiedemann et al., 1998). Some studies have been performed with TFMKs and electrophysiological assays in *Plutella xylostella* (Prestwich and Streinz, 1988), *Thaumetopoea pityocampa* (Parrilla and Guerrero, 1994), *M. brassicae* (Renou et al., 2002), *Cydia pomonella* (Giner et al., 2009), and *S. frugiperda* (Malo et al., 2013). Moreover, TFMKs have been used with antennal extracts of different moth species, e.g., *S. littoralis* (Durán et al., 1993), *Ostrinia nubilalis* (Riba et al., 2005), and *C. pomonella* (Giner et al., 2009). All of these studies have shown that TFMKs had an effect on the catalytic activity of antennal esterases. On the other hand, behavior assays in tunnel wind with these inhibitors have shown a disruptive effect on the orientation flight in males of *S. nonagrioides*, *S. littoralis*, and *C. pomonella* (Bau et al., 1999; Giner et al., 2009). In the field, it has been reported that TFMKs are effective pheromone antagonists for several insect pests, such as *S. nonagrioides* (Riba et al., 2001), *C. pomonella* (Giner et al., 2009), *Zeuzera pyrina* (Muñoz et al., 2011), *O. nubilalis* (Solé et al., 2008), or *T. absoluta* (Dominguez et al., 2016). The reduction in damage caused by *S. nonagrioides* and *O. nubilalis* in maize fields after application of Z11-16:TFMK was particularly remarkable, i.e., this is an analogue of the pheromone of the former species at a dose of 80 g/ha (Solé et al., 2008).

Riba et al. (2001) further evaluated the biological toxicity of (*Z*)-11-hexadecenyl TFMK (Z11-16:TFMK) and 3-octylthio-1,1,1-trifluoropropan-2-one (OTFP) in mice, and the results showed low toxicity (LD_50_ 1 g/kg after the 6th day of the assay). Overall, an accurate identification and characterization of these enzymes could provide the basis for the development of putative inhibitory compounds that will be an alternative to IPM.

## Concluding Remarks and Further Perspectives

The olfactory system is critical for reproductive success in many insect species. Some of these are of great economic importance in agriculture and forestry fields such as moths from the Tortricidae and Pyralidae family. Fortunately, the semiochemical compounds involved in host recognition, mating, or defense behaviors are being used to manage insect pests through environmentally friendly approaches, e.g., mating disruption and mass trapping. However, globalization has facilitated the dissemination of insect species throughout the world. How do insects adapt to these new environments? More specifically, how their olfactory system responds to these new conditions?

To answer these questions, it is important to understand the molecular basis and mechanisms involved in insect olfaction where proteins are the main players. Comprehensive studies have been performed for ORs, OBPs, and CSPs; ODEs have garnered less attention. These enzymes are novel targets for the use of species-specific chemicals in IPM. Therefore, we propose that an improved approach to classify a certain ODE into PDE would be crucial based on a highly specific sex pheromone communication. The evidence presented in this work suggests that a limited number of ODEs would actually be antennal- and pheromone-specific. Therefore, gene expression through RT-qPCR should consider comparing between sexes in antennae and then in the rest of tissues.

Phylogenetic analyses could help to filter ODEs close to already characterized PDEs (e.g., ApolPDE). Particularly, some studies (*S. inferens* and *S. littura*) found that a monophyletic clade (proposed as PDE clade) is present in moths. With this in mind, further ODEs that fall within this clade could be considered for characterization as putative PDEs. Finally, heterologous expression of the selected ODE(s) with purification and kinetic assays would be crucial for such task.

Alternatively, localization techniques such as FISH in specific sensilla could strongly support pheromone degradation function. Of note, transcriptomics has arisen as a useful tool to identify putative enzymes. Four modeled structures (ApolPDE1, SinfCXE13, SlitCXE13, and EsemCXE6) were shown to visualize their conformation and the residues of the active site, which resulted in conserved function. With the above issue addressed, the identification and characterization of these enzymes could provide the basis for the development of putative inhibitory compounds (e.g., TFMKs) that will be a complement to IMP strategies.

## Author’s Note

All the information performed in this work is of a public nature. We created the figures and the data generated in relation to the transcripts identified from the antennal transcriptome of *E. semipurpurella*.

## Author Contributions

RG wrote all sections. JM developed the tables and figures. HV and AM conceived the idea for the review article. AQ supervised the project. All authors contributed to the article and approved the submitted version.

### Conflict of Interest

The authors declare that the research was conducted in the absence of any commercial or financial relationships that could be construed as a potential conflict of interest. All the information performed in this work is of a public nature. We created the figures and the data generated in relation to the transcripts identified from the antennal transcriptome of *E. semipurpurella*.
