# Supplementary material for: An Overview of Antennal Esterases in Lepidoptera
Source: Front Physiol. 2021 Mar 31;12:643281. doi: 10.3389/fphys.2021.643281 (PMC8044547; doi:10.3389/fphys.2021.643281)
Supplement: Supplementary file 1 [file Table_1.DOCX]

**Supplementary Material**

**Figure S1. Phylogenetic tree of esterases.** 89 esterases sequences were used to perform the phylogenetic tree. Esterases from *Spodoptera littoralis*, *S. litura*, *S. exigua*, *Sesamia inferens*, *Antheraea polyphemus*, *Eriocrania semipurpurella*, *Popillia japonica* and *Drosophila melanogaster*. Monophyletic clade in red represents the PDE clade. Phylogenetic analyses were performed by using MAFFT sever for multiple sequence alignments and FastTree software for phylogenetic relationships based on maximum-likelihood method (Price et al., 2010).
