# Supplementary figures and images for: An Overview of Antennal Esterases in Lepidoptera
Source: Front Physiol. 2021 Mar 31;12:643281. doi: 10.3389/fphys.2021.643281 (PMC8044547; doi:10.3389/fphys.2021.643281)

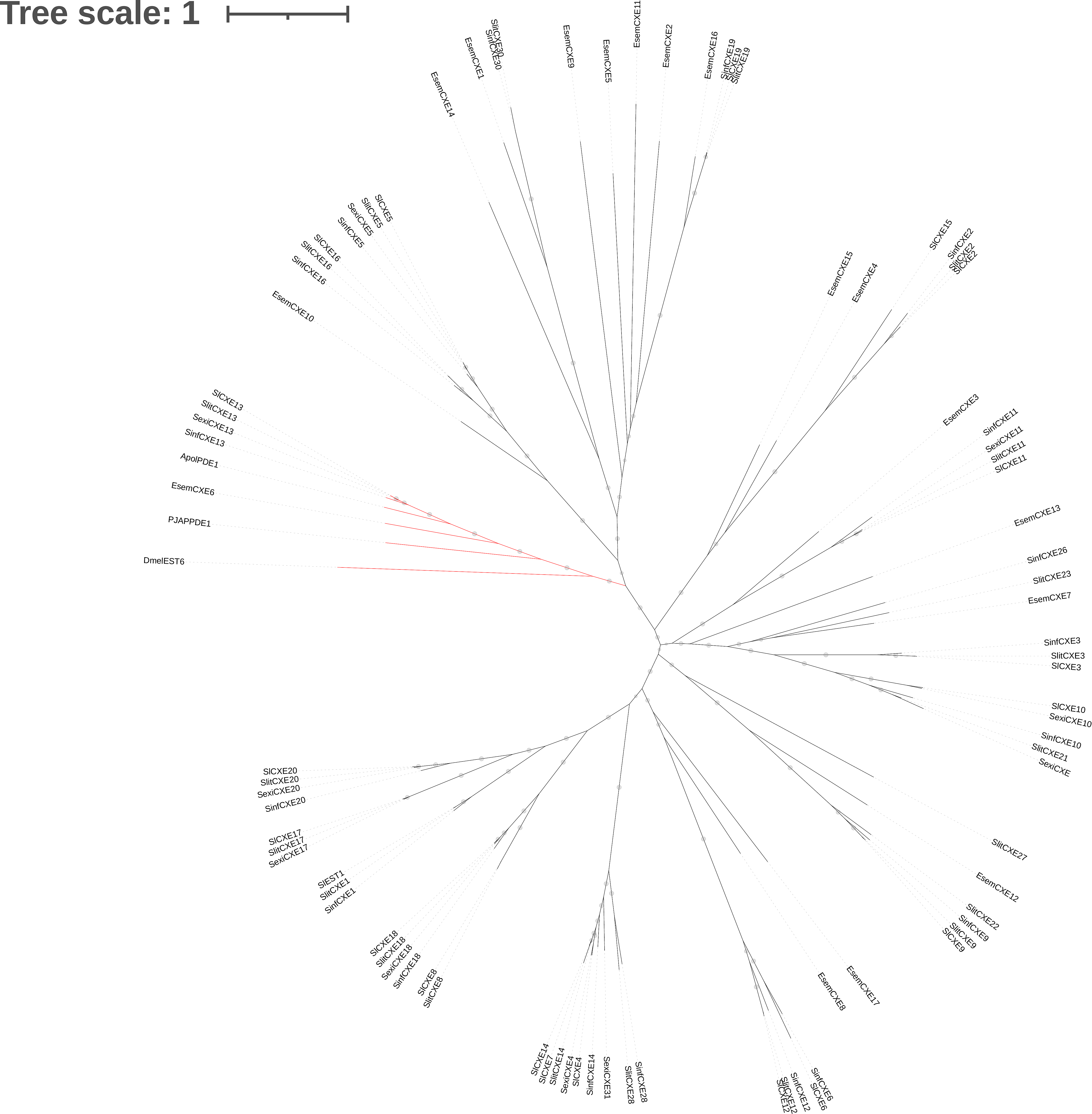

Supplement: Supplementary file 2 [file Image_1.TIFF]
